# Stable and unstable associations between learning environment factors and study approaches: two consecutive cross-sectional analyses of Norwegian occupational therapy students

**DOI:** 10.1007/s10984-022-09445-7

**Published:** 2022-12-24

**Authors:** Gry Mørk, Linda Stigen, Astrid Gramstad, Trine A. Magne, Tove Carstensen, Tore Bonsaksen

**Affiliations:** 1 Department of Health, Faculty of Health Studies, VID Specialized University, Stavanger, Norway; 2 Faculty of Health and Function, Western Norway University of Applied Sciences, Bergen, Norway; 3 Norwegian University of Science and Technology (NTNU), Gjøvik, Norway; 4 UiT – The Arctic University of Norway, Tromsö, Norway; 5 Centre for Care Research North, Tromsö, Norway; 6 Norwegian University of Science and Technology (NTNU), Trondheim, Norway; 7 Department of Health and Nursing Sciences, Faculty of Social and Health Sciences, Inland Norway University of Applied Sciences, Elverum, Norway

**Keywords:** Approaches to learning, Entwistle model, Generic skills, Higher education, Learning environment, Occupational therapy

## Abstract

Relationships between learning environment variables and students’ approaches to studying have been investigated from many points of view over the last decades. However, few studies have explored whether such relationships are stable over time. In the two consecutive cross-sectional analyses performed in this study, Norwegian occupational therapy students’ perceptions of their learning environment and their approaches to studying were assessed in the second (162 students) and third (193 students) year of their study program. Aside from sociodemographic information, the students completed the Course Experience Questionnaire and the Approaches and Study Skills Inventory for Students, with the aim of exploring whether associations between learning environment variables and study approaches were stable across time. The data were analyzed with hierarchical linear regression analyses. Relatively stable associations with students’ study approaches were found for the learning environment variables of ‘generic skills’ and ‘appropriate workload’. The learning environment variables of ‘clear goals and standards’ and ‘student autonomy’ were directly associated with study approaches in both study years, but the nature of the associations shifted during the study period. Thus, knowledge of stability and change in these relationships could assist faculty in promoting a well-functioning learning environment throughout the study program.

## Introduction

The learning environment is the social, psychological and pedagogical context in which learning occurs, and it is believed to affect student achievement and attitudes (Fraser, 1998). Entwistle (2018) further described the learning environment as a whole range of influences on student learning; not only the teaching, but also the broader context that shapes students’ way of studying. These include, for example, the students’ perceptions of goals and standards, the workload, and whether the study program facilitates development of generic skills and student autonomy. Pekrun and coauthors (2009) pointed out that the students’ perceptions of the learning environment also concern their achievement goals and achievement emotions. How students’ approach or avoid performance predicts achievement emotions, such as enjoyment, boredom, anger, hope, pride, anxiety, hopelessness, and shame. In turn, students’ emotions predict students’ performance (Pekrun et al., 2009). The faculty’s responsibility is to provide a learning environment that encourages the student to perform the necessary learning activities, and to assess student performances against the intended learning outcomes (Biggs & Tang, 2011). According to Pekrun and co-workers (2009), this also helps to facilitate and support students’ competence and overall psychological functioning in the learning environment.

Students should be able to reproduce knowledge, but also acquire a personal, reflective, and critical relationship to one’s own knowledge, which is described as general competences: the *ability to use knowledge and skills in an independent manner in different situations by demonstrating the ability to cooperate, responsibility, and a capacity for reflection and critical thinking, in educational and work contexts* (Ministry of Education & Research, 2014, p. 5)*.* Developing generic skills is crucial, not only in the university and professional context, but also for lifelong learning. Studies from different fields have shown that required professional skills, such as social skills, organizing skills, skills for knowledge acquisition, and problem-solving skills, are not only field- or profession-specific but also generic (Chan & Fong, 2018; Ebekozien et al., 2022; Tynjälä et al., 2016).

Following years of revision, Biggs proposed *the 3P model* as a systematic learning process model (Biggs & Moore, 1993). The three Ps in the model refer to presage, process, and product. The model proposes that personal characteristics and environmental influences (presage factors) combine to create the approach that students use in their learning (process factors), which in turn influences student performance on assessments (product factors). The learning approaches in this 3P model, the deep and the surface approach, are described by Biggs (1999) through the well-known narrative about ‘Susan’ and ‘Robert’. The narrative is not meant to describe different personalities, but rather how these two archetype students usually go about their studying. Susan is characterized by a preference for deep learning (Marton & Säljö, 1976). She reflects on possibilities, implications, applications, and consequences and likes to get to the bottom of things to reach understanding. Conversely, Robert doesn’t really care about the learning process and sticks with lower-level learning activities such as note-taking without relating and theorizing, and he tries to memorize, hopefully with sufficient effort to pass examinations and obtain a qualification for a job. According to Biggs (1999), he might not even be studying in his preferred area. Robert appears to adopt a surface approach to learning (Marton & Säljö, 1976).

In Ramsden and Entwistle’s (1981) research on study approaches, a third strategic approach was included. The researchers were the first to establish statistical relationships between approaches to studying and students’ perceptions of the learning environment. In their study, the meaning orientation (synonymous to the later-developed concept ‘deep approach’) was associated with perceptions of good teaching and student autonomy, while the reproducing orientation (synonymous to the later-developed concept ‘surface approach’) was associated with perceptions of having a heavy workload and lack of student autonomy (Ramsden & Entwistle, 1981). The third approach, the strategic approach to studying, refers to students preoccupied with managing and organizing their studies and their time (Entwistle & McCune, 2004). Students using a strategic approach often identify with the aim of obtaining the best possible examination results through well-organized studying and alertness to assessment requirements. Some students are more influenced by the environment than others (Nijhuis et al., 2008). Students’ inclination to use the strategic approach seems to be relatively stable over time, compared with the deep and surface approaches which can be more amenable to change (Mørk et al., 2022; Postareff et al., 2018).

Biggs’ 3P model has been criticized for a lack of definition of the underlying structure (Howie & Bagnall, 2013). de la Fuente and co-workers integrated the variables of externally-regulated learning and self-regulated learning in the context of Biggs’ 3P model (de la Fuente, 2017)*. Self-regulated learning* is a cyclical process wherein students plan for a task, monitor their performance, and then reflect on the outcome. *Externally-regulated learning,* on the other hand, refers to situations in which students depend on the teacher’s guidance and control, or a textbook, or classmates, to regulate learning processes*.* Integrating these variables into the 3P model, de la Fuente (2017) illustrated that, during any teaching–learning process, different levels of student self-regulation (low-medium–high) occur in combination with different levels (low-medium–high) of regulatory teaching. This allows an understanding of how students’ levels of self-regulated learning vary between contexts of interaction.

Over the last decades, research reports have shown that the combination of the two factors (*self-regulation* and *external-regulation*) determines the cognitive-strategic factors underpinning learning motivation (i.e. the student’s learning approach, de la Fuente et al., 2022). Further, relationships between learning approaches and learning outcomes have been widely examined in higher-education contexts. For example, studies of the relationships between approaches to studying and general competences have revealed that a deep approach to studying is positively related to general competences, whereas a surface approach is negatively related to general competences (Kreber, 2003; Liu et al., 2015; Lizzio et al., 2002).

Longitudinal studies of students’ study approaches and perceptions of the learning environment have shown different results in recent decades (Asikainen et al., 2014; Ballantine et al., 2008; Hall et al., 2004; Jackling, 2005; Wierstra et al., 2003). In a study of international business students in the Netherlands, learning environment variables such as clear goals, appropriate workload, and independent learning correlated significantly with deep approach to learning, while the perception of a heavy workload was significantly related to the surface approach to learning (Nijhuis et al., 2008). In a study of health sciences students, a change in the curriculum resulted in a small but significant increase in deep and strategic approach over time and a decrease in the surface approach (Walker et al., 2010). Nevertheless, Gijbels and coworkers (2009) found that changes deliberately implemented in the learning environment did not correspond with desired changes in students’ study approaches. Some researchers even suggest that any changes in students’ study approaches are more strongly related to individual elements than to contextual elements (Postareff et al., 2014).

A national inquiry in Norway has so far expanded the knowledge about occupational therapy students’ perceptions of the learning environment (Stigen et al., 2022; Thordardottir et al., 2020) and approaches to studying (Gramstad et al., 2020; Mørk et al., 2020, 2022; Thørrisen et al., 2020) during the three years of study. One of these studies constitutes a particularly interesting backdrop for this study, because it examined associations between perceptions of the learning environment and approaches to studying among the students while in their first year of the study program (Mørk et al., 2020). However, because that study only included one measurement, the degree to which these associations are stable over time is not known. If associations are found to vary over time, knowledge about phase of study could improve understanding of how learning environment factors can influence students’ study approaches.

### Study aim

The aims of this study were to examine learning environment variables associated with occupational therapy students’ approaches to studying while in their second and third study years and to evaluate whether associations are stable across time.

## Method

### Research design and study context

This study is part of an investigation of occupational therapy students’ perceptions of the learning environment and approaches to studying. Students enrolled in each of the six occupational therapy programs in Norway participated, and these three-year Bachelor’s degree programs had class sizes between 24 and 77 students. The duration of the program is three years, with a minimum of 1/4 of the study program (i.e. 5–6 months in total) being clinical practice studies (Thordardottir et al., 2020). The occupational therapy students typically participated in diverse study activities, such as traditional lectures, seminars, case studies, problem-based, team-based, project-based learning, and individual self-organized studies.

### Procedure, participants and response rate

At each of the education programs, one member of faculty distributed the questionnaires and consent forms to students. The only inclusion criterion was being a member of the targeted cohort of occupational therapy students. There were no exclusion criteria. After the data collection in the first year of study, student cohorts were followed-up with an annual survey in the second and third years of study, with collection of data within the time intervals of December 2018–February 2019 and December 2019–February 2020, respectively. The questionnaires were identical in all three study years. This study employed cross-sectional data from students in their second and third year of the study program.

Overall, 305 students where eligible to participate from the six educational programs. In the second year, 168 students participated (55.1% response rate) while 200 students participated the third year (response rate 65.6%). Students with missing values on one or more variables were removed from the analyses, resulting in a sample sizes of 162 participants in the second year and 193 participants in the third year. The collected data were from a single cohort of students, which implies that year 2 was year 2 students and year 3 was year 3 students.

### Measurements

#### Sociodemographic variables

Continuous data on age (years) and time spent on independent studying (average hours per week) were collected. The study also registered categorical variables of gender (male = 0, female = 1), having prior experience from higher education (no = 0, yes = 1), and having occupational therapy as the highest prioritized line of education at the time of enrolment (no = 0, yes = 1).

#### Learning environment

The *Course Experience Questionnaire* (CEQ) was used to measure aspects of the learning environment. This questionnaire focuses on students’ summative experience at the study program level, rather than focusing on individual subjects or teachers. The extended CEQ (Wilson et al., 1997) consists of 37 items using a five-point rating scale (strongly agree to strongly disagree). One of the items assesses overall satisfaction with the course. The extended CEQ has six subscales that address issues of clear goals and standards, emphasis on independence, good teaching, appropriate workload, appropriate assessment, and generic skills. The validated Norwegian translation of this version (Pettersen, 2007) was used in the current study. Previous exploratory and confirmatory factor analyses of large multidisciplinary samples of students and graduates from several universities have established both the reliability and the structural validity of both the full and short forms of the instrument (Wilson et al., 1997). In the current study, the sample size was insufficient for conducting a factor analysis given the large number of items on the CEQ; thus, only reliability estimates were established. With first year students, internal consistency of the scales was 0.73 (clear goals and standards), 0.63 (emphasis on independence), 0.70 (good teaching), 0.69 (appropriate workload), 0.45 (appropriate assessment), and 0.83 (generic skills) (Mørk et al., 2020; Thordardottir et al., 2020). Considering the internal consistency results, the ‘appropriate assessment’ scale was not included in subsequent analyses (Bonsaksen et al., 2019). Table 1 displays scales and sample items from the CEQ.

**Table 1:** Scales and example items from the *Course Experience Questionnaire*

Scale	Sample item
Clear goals and standards	The aims and objectives of this course are not made very clear*
Student autonomy	Students have a great deal of choice over how they are going to learn in this course
Good teaching	The staff make a real effort to understand difficulties students may be having with their work
Appropriate workload	The sheer volume of work to be got through in this course means you can't comprehend it all thoroughly*
Generic skills	This course has helped me develop the ability to plan my own work

The scale ‘Appropriate assessment’ was excluded from the current study

*This item has reversed coding

#### Approaches to studying

To measure the students’ study approaches, we used the *Approaches and Study Skills Inventory for Students* (ASSIST) (Entwistle et al., 2013) or, more specifically, a validated Norwegian translation of the inventory (Diseth, 2001). The ASSIST consists of 52 statements about what students usually do in study and learning situations. Participants are instructed to rate their level of agreement (1 = disagree, 2 = disagree somewhat, 3 = unsure, 4 = agree somewhat, 5 = agree). The inventory has a three-factor structure, which was replicated in a cross-cultural study of undergraduate occupational therapy students (Bonsaksen et al., 2019b) and in the current sample (Dalomba et al., 2020). All subscales were found to load on the theoretically-proposed main scales (Dalomba et al., 2020). The main scale scores for *deep, strategic,* and *surface* approaches to studying are calculated by adding the scores on the relevant items. When used with the sample while in their first year of study, the internal consistency estimates (Cronbach’s α) for the study approach scales were 0.71 (deep approach), 0.84 (strategic approach), and 0.76 (surface approach) (Dalomba et al., 2020; Mørk et al., 2020). Table 2 displays the scales and sample items from the ASSIST.

**Table 2:** Scales and sample items from the Approaches and Study Skills Inventory for Students

Scale	Sample item
Deep approach	I try to relate ideas I come across to those in other topics or other courses whenever possible When I have finished a piece of work, I check it through to see if it really meets the requirements
Strategic approach	I think I'm quite systematic and organised when it comes to revising for exams I look carefully at tutors’ comments on course work to see how to get higher marks next time
Surface approach	I’m not really interested in this course, but I have to take it for other reasons I like to be told precisely what to do in essays or other assignments

### Data analysis

The study design comprised two consecutive cross-sectional analyses. The sample of the second and third year were described with means and standard deviations on continuous variables and with frequencies and percentages on categorical variables. Two consecutive hierarchical linear regression analyses were performed, using the deep, strategic and surface approach scales as outcome variables. Independent variables were included in two subsequent blocks, with the first block representing the sociodemographic factors (age, gender, time spent on independent study, educational priority, and prior higher education) and the second block representing learning environment factors (clear goals and standards, student autonomy, good teaching, appropriate workload, and generic skills). Effect sizes were reported as standardized *β* coefficients and statistical significance was set at *p* < 0.05. The analyses were performed using IBM SPSS Statistics (Version 26).

### Research ethics

All participants provided written informed consent to participate in this study. Approval for collecting, storing, and using the data was granted on October 12, 2017, by the Norwegian Center for Research Data (Project No. 55875).

## Results

### Sample characteristics

Table 3 displays background characteristics, perceptions of the learning environment, and approaches to studying for the sample of occupational therapy students in their second and third year of study.

**Table 3:** Sample in the second and third study years: descriptive statistics

Variables	Study year	
	2nd year (*n* = 162)	3rd year (*n* = 193)
	M (SD)	M (SD)
*Sociodemographic variables*		
Age at enrolment (years)	22.4 (4.1)	22. 7 (4.5)
Time spent on independent study (hours)	9.2 (6.8)	8.4 (6.7)
	n (%)	n (%)
Female gender	130 (80.2)	149 (77.2)
Priority line of study	101 (62.3)	126 (65.3)
Prior higher education	63 (38.9)	78 (40.4)
*Learning environment*		
Clear goals and standards	17.0 (3.2)	17.0 (3.6)
Student autonomy	18.1 (3.8)	18.0 (4.6)
Good teaching	25.2 (5.3)	26.0 (6.1)
Appropriate workload	15.4 (3.7)	15.2 (3.9)
Generic skills	23.7 (3.1)	24.6 (4.6)
*Approaches to studying*		
Deep approach	57.3 (7.7)	57.5 (7.9)
Strategic approach	72.2 (9.8)	72.1 (9.1)
Surface approach	44.9 (8.5)	44.8 (9.6)

### Adjusted associations with the study approach scales

Table 4 displays results from the regression analysis for students in the second and third study year, with students’ scores on the deep, strategic, and surface study approaches as dependent variables.

**Table 4:** Associations of sociodemographic and learning environment variables with study approach scores in the second and third study years

Variables	2nd study year (*n* = 162)			3rd study year (*n* = 193)		
	Deep	Strategic	Surface	Deep	Strategic	Surface
*Sociodemographic variables*						
Age at enrolment	0.17*	− 0.03	− 0.14	0.28***	− 0.03	− 0.18**
Female gender	− 0.06	0.17*	0.08	− 0.05	0.11	− 0.01
Time spent on independent study	0.18*	0.27***	− 0.03	0.18**	0.22**	0.04
Priority line of study	− 0.08	− 0.03	− 0.06	− 0.08	0.01	− 0.13*
Prior higher education	0.17*	0.08	0.08	0.12	− 0.02	− 0.02
Explained variance	11.3%**	11.0%**	**6.4%**	15.8%***	7.3%*	7.1%*
*Learning environment*						
Clear goals and standards	− 0.00	0.10	− 0.23*	0.09	0.21**	− 0.10
Student autonomy	− 0.02	0.08	0.20*	− 0.05	0.23**	− 0.03
Good teaching	0.09	− 0.00	− 0.06	0.14	− 0.05	− 0.06
Appropriate workload	0.12	− 0.14	− 0.31***	0.11	0.03	− 0.45***
Generic skills	0.29**	0.15	− 0.16	0.20**	0.20**	− 0.01
*R*^2^ change	13.2%***	6.9%*	21.6%***	13.3%***	19.3%***	25.9%***
Explained variance	24.6%***	17.8%**	28.0%***	29.1%***	26.5%***	33.0%***

Bold is used to differentiate between beta values (not bold) and percentage explained variance/R2 change (bold)

^*^p < 0.05, **p < 0.01, ***p < 0.001

In the second year of study, higher age (*β* = 0.17, *p* < 0.05), spending more time on independent study (*β* = 0.18, *p* < 0.05), having prior higher education (*β* = 0.17, *p* < 0.05) and having a stronger sense of developing generic skills during the study program (*β* = 0.29, *p* < 0.01) were directly associated with higher deep approach scores. Being female (*β* = 0.17, *p* < 0.05) and spending more time on independent study (*β* = 0.27, *p* < 0.001) were directly associated with higher strategic approach scores. Perceiving goals and standards as unclear (*β* = − 0.23, *p* < 0.05), as well as perceiving the study program as emphasizing student autonomy (*β* = 0.20, *p* < 0.05) and the workload to being too high (*β* = − 0.31, *p* < 0.001), were directly associated with higher surface approach scores.

In the third year of study higher, age (*β* = 0.28, *p* < 0.001), spending more time on independent study (*β* = 0.18, *p* < 0.01) and having a stronger sense of developing generic skills during the study program (*β* = 0.20, *p* < 0.01) were directly associated with higher deep approach scores. Spending more time on independent study (*β* = 0.22, *p* < 0.01), perceiving the goals and standards to be clear (*β* = 0.21, *p* < 0.01), perceiving the study program to emphasize student autonomy (*β* = 0.23, *p* < 0.01) and having a stronger sense of developing generic skills during the study program (*β* = 0.20, *p* < 0.01) were directly associated with higher strategic approach scores. Lower age (*β* = − 0.18, *p* < 0.01), not having occupational therapy as the first priority line of study at the time of enrolment (*β* = − 0.13, *p* < 0.05) and perceiving the workload to be too high (*β* = − 0.45, *p* < 0.001) were directly associated with higher surface approach scores.

## Discussion

The aims of this study were to examine learning environment variables associated with occupational therapy students’ approaches to studying while in their second and third study years and to evaluate whether associations were stable across time. Relatively stable associations between the perceived learning environment and students’ study approaches were found for generic skills and appropriate workload. The variables clear goals and standards and student autonomy were directly associated with study approaches in both study years, but the nature of the associations shifted during the study period.

During the study program, generic skills showed stable associations with the study approach scales. In the Course Experience Questionnaire, the generic skills scale includes development of problem-solving skills, analytic skills, teamwork, and the ability to plan work (Lizzio et al., 2002; Pettersen, 2007). Stable associations were found between having a stronger sense of developing generic skills and higher ratings on the deep approach to studying both in the second and third year of study. This association was also detected in the study of first-year students (Mørk et al., 2020). The prior study also found that students with higher scores on generic skills were more inclined to have higher scores on the strategic approach scale and, conversely, lower scores on generic skills were associated with higher surface approach scale scores (Mørk et al., 2020). The association between strategic approach and generic skills was also present in the third year of study. In the second year of study, this association was not significant, but it had the same direction as in the first and third study years. The findings represent stability over time in the association between generic skills and study approaches.

The associations between generic skills and study approaches are in line with findings reported by Kreber (2003) and Tuononen et al. (2020), especially highlighting that the deep approach to studying having the strongest relationship with generic skills. Liu and coworkers (2015) also found a positive correlation between self-perceived overall competence and deep approach and a negative correlation between surface approach and self-perceived overall competence. Hall et al. (2004) found that the introduction of problem-solving exercises in collaborative groupwork was related to an increase in students’ deep approach to studying as well as to a decrease in students’ surface approach to studying. Virtanen and Tynjälä (2018) suggested facilitating a student active learning environment, because the results of their research showed that the traditional forms of university teaching and studying, such as reading, lecturing and working alone, correlated negatively with the acquisition of generic skills.

Self-regulated learning comprises some of the same abilities as reflected in concept generic skills: a personalized, reflective, and critical relationship to one’s own knowledge. In a study of personal self-regulation, learning approaches, resilience and test anxiety, the questions used to assess self-regulation involved goal-setting, decision-making and learning from mistakes (de la Fuente et al., 2017). They would be equally relevant for assessing generic skills as described in the CEQ (Wilson et al., 1997). In their study, de la Fuente and coworkers (2017) found a significant positive association between self-regulation and the deep approach, and a significant negative correlation between self-regulation and the surface approach. Further, this led the researchers to consider that self-regulation can be a presage factor (according to Biggs 3P Model), and that levels of self-regulation to a certain extent determine students’ adoption of a learning approach (de la Fuente et al., 2017). In view of the above, our results appear to largely support the associations detected in previous studies, while also adding to the knowledge base by establishing a high degree of stability over time in the relation between generic skills and the study approach scales.

An inappropriate workload showed a stable association with higher ratings on the surface approach to studying in both the second and third year, which mirrors the results from the first year of study (Mørk et al., 2020). Because the relationship between high perceived workload and a surface study approach is one of the most consistent findings in the field (Kember et al., 1995; Kreber, 2003; Lizzio et al., 2002), it is no surprise that students who found the workload to be too high preferred an approach to studying involving shortcuts to cope with the study situation. On a similar note, de la Fuente and coworkers (2017) found that students with a deep approach to studying were tenacious, had a perception of control, managed stress well and adapted to change, while students with a surface approach did not have these characteristics. If this is the case, students using the surface approach would easily perceive the workload as too high. Resilience among students therefore could predict the type of approach that students will use when studying in higher education (de la Fuente et al., 2017b). This in line with Diseth’s consideration that ‘appropriate workload’ could have a less obvious relationship with the learning environment, because “any given amount of work that may be appropriate for one particular student may represent an overload of work to another” (Diseth, 2007, p. 384). Thus, while the relationship between perceptions of a heavy workload and a surface approach to studying was found to be stable across years of study, the working mechanism behind this relationship needs closer scrutiny, because it might be linked to individual elements (the student) as well as to contextual elements (the learning environment).

The results showed that students perceiving the goals and standards to be unclear was directly associated with higher surface approach scores in the second year of study. This is consistent with findings from the study of first year students (Mørk et al., 2020). In the third year, perceiving the goals and standards to be clear became directly associated with the strategic approach. According to Biggs (2001), students using the strategic approach are well-organized and have an alertness to assessment requirements. The finding in our study makes it possible to speculate if the students gradually adapt to the given goals and standards and learn how to study strategically towards the goals and standards in the course in order to produce the best possible examinations results. To facilitate a learning environment that stimulates productive approaches to learning, educators should prioritize to clarify the goals and standards and, if necessary, discuss the goals with students to avoid confusion.

Wolters (2004) suggested that students who do not clearly see the goal structures in the classroom more often procrastinate and disengage from learning tasks when experiencing difficulties or boredom. One way to clarify the goals and standards is to communicate lecture goals to students, as well as afterwards reflecting with the students on whether these goals were achieved (Liborius et al., 2019). These researchers emphasized that students benefit from setting goals and planning their study day and that, in that way, they avoid procrastination. Pekrun and co-workers (2009) pointed out that students’ performance-approach goals positively predicted pride and hope, whereas performance-avoidance goals predicted anxiety, hopelessness and shame. This also connects with de la Fuentes’ (2017) theory of self-regulated versus externally-regulated learning, in which goals and standards are relevant external factors that contribute to regulate the learning process. The level of regulatory teaching, as an external regulatory variable, has been shown to be associated with academic emotions in learners (de la Fuente et al., 2016). When the academic context is predictable (e.g. by having established clear goals and standards), students might experience lower levels of stress and less negative emotions. Our results appear to support the associations reported in previous studies, especially with regards to stability over time in the relation between clear goals and standards and productive study approaches.

We found associations between student autonomy and the approaches to studying, which did not occur the first year. The findings shifted from a correlation between higher student autonomy and higher surface approach in the second year, to higher student autonomy and higher strategic approach in the third year. Possibly, this could be a result of their increased maturity and their adapting to the student role and the tasks and expectations in the education program (Thordardottir et al., 2020). When learning environments provide the opportunity for self-regulated learning and student autonomy, students can develop self-regulation abilities. Students’ autonomy and increased control over their own learning process both require and support self-direction competencies (de la Fuente et al., 2016). Thus, adapting to the student role and developing self-regulation abilities can be conceived as a process in which responsibility for one's own learning shifts from being (too big) a challenge to becoming an advantage for the students.

### Sociodemographic covariates to study approaches

Stable associations were found between spending more time on independent study with higher ratings on the strategic approach in all three study years, and with higher deep approach ratings in the second and third year. Whether time spent on independent study leads to a more-productive study approach, or whether the approach itself leads to spending more time studying independently, is uncertain. Cyclical, self-strengthening associations are viable. The relationship between higher age and higher levels of deep approach to studying was detected in all three years of the study. In this study, we also found an association between lower age and higher surface approach ratings in the third year, and this result was in the same direction in the first and second years of study, although not statistically significant. Zeegers (2001) argued that younger students, in particular in the first year of study, adopt survival strategies relating to a surface approach, while older students are more likely to adopt productive study strategies and therefore are more successful. Our results regarding sociodemographic covariates to study approaches suggest a high degree of stability over time in the way in which higher age and more time spent on independent studying are associated with productive study approaches.

Having prior higher education was associated with higher ratings on the deep approach to studying in the first and second years of study. This could indicate that prior academic experience is beneficial for students entering the occupational therapy study program, but that the early benefit is reduced over time as all students gain more academic experience during their ongoing study. The study of first-year students revealed that female students had higher ratings on the strategic approach to studying (Mørk et al., 2020), with this association also being found in the second year of study. Another stable association was that students who did not have occupational therapy as the first priority line of study were more inclined to use the surface approach to studying in the first (Mørk et al., 2020) and in the third year of study. It is noteworthy that this finding was still significant after three years, when the students have had a long time to process the fact that they were not admitted to their preferred line of education.

### Study’s strengths and limitations

The inclusion of all the six occupational therapy programs in Norway increased the validity of the results obtained in this study. However, the response rates between the different programs differed and could have led to sample bias. It is possible that the length of the survey (111 questions) discouraged participation among some students, as suggested by Entwistle and McCune (2004). The study was limited by using only one group of healthcare students. The study employed surveys in each of the three study years, which allowe evaluating whether associations were similar or different between years of study. However, because of the cross-sectional nature of the data collected, the direction of the associations (e.g. cause and effect) cannot be established by our results. A strength of the study is also our use of comprehensive and validated instruments for measuring learning environment factors and study approaches. However, the current study sample was too small for formally examining the structural validity of the CEQ. Because data collection was completed before the onset of the Covid-19 pandemic, the results of the study are unrelated to the specific circumstances experienced by students during the pandemic.

### Conclusion and implications

This study suggests that associations between learning environment variables and students’ approaches to studying are stable over time. Our results show that associations were mostly similar over the course of the full study program, while the relationship between student autonomy and study approaches could change over time, possibly because of increased student maturity. Thus, while more faculty structure and leadership could be needed during the early years in the study program, more student autonomy might be beneficial during the later phases of the study program to sustain students’ use of deep and strategic study approaches. Our findings support the perspectives in the theory of self-regulated versus externally regulated learning. A suitable learning environment might serve as a positive external regulator that promotes autonomy while students progress from lower to higher levels of self-regulated learning.

Facilitating a positive learning environment in occupational therapy education could have an impact on students’ approaches to studying. A positive learning environment in part can hinge on students’ understanding of the importance of general competences in relevance to future work so that they are motivated to develop these competences during their studies. Students at risk of adopting a surface approach can possibly benefit from improving their self-regulation in learning situations. Furthermore, academic staff can support students in their studying by making changes in the curriculum to ensure that the workload is appropriate, and that the goals and standards in courses are clearly communicated to the students.
